# Molluscicidal Screening of *Hypocreales* Fungi from a Brazilian Cerrado Cave Against *Biomphalaria glabrata* Snails

**DOI:** 10.3390/jof11030173

**Published:** 2025-02-21

**Authors:** Dominnyke Slater Santos Neves, Cyntia Ayumi Yokota Harayashiki, Pedro Henrique Félix de Oliveira, Thiago Lopes Rocha, Jadson Diogo Pereira Bezerra

**Affiliations:** 1 Programa de Pós-Graduação em Biologia da Relação Parasito-Hospedeiro (PPGBRPH), Instituto de Patologia Tropical e Saúde Pública, Universidade Federal de Goiás, Goiânia 74605-050, GO, Brazil; dominnyke.slater@discente.ufg.br (D.S.S.N.); felix.pedro@discente.ufg.br (P.H.F.d.O.); 2 Laboratório de Micologia, Departamento de Biociências e Tecnologia, Instituto de Patologia Tropical e Saúde Pública, Universidade Federal de Goiás, Goiânia 74605-050, GO, Brazil; 3 Laboratório de Biotecnologia Ambiental e Ecotoxicologia, Departamento de Biociências e Tecnologia, Instituto de Patologia Tropical e Saúde Pública, Universidade Federal de Goiás, Goiânia 74605-050, GO, Brazil; c.harayashiki@gmail.com (C.A.Y.H.); thiagorochabio20@ufg.br (T.L.R.)

**Keywords:** biotechnological potential, cave mycodiversity, fungal bioprospecting, fungal filtrate, gastropod control

## Abstract

Fungi play vital roles in ecosystems through parasitism, commensalism, and mutualism. Additionally, they are widely used in industry as bioactive compound producers and biological control agents. *Biomphalaria glabrata* is a freshwater snail often controlled with chemical molluscicides. However, developing effective alternatives to these chemical treatments is essential. This study evaluated the molluscicidal potential of culture supernatant from *Hypocreales* fungi isolated from a cave in the Brazilian Cerrado against the *B*. *glabrata*. The isolates were identified based on morphological features and ITS rDNA sequences. Fifteen filtrates of *Hypocreales* fungi were obtained and tested both pure and in different dilutions (10% and 50%) against newly hatched snails during 96 h of exposure. The fungal isolates were identified as belonging to the genera of *Clonostachys* (1), *Cylindrocladiella* (1), *Fusarium* (1), *Gliocladiopsis* (1), *Keithomyces* (1), *Marquandomyces* (1), *Ovicillium* (1), *Pochonia* (1), *Purpureocillium* (1), *Sarcopodium* (1), *Sarocladium* (1), *Trichoderma* (3), and *Volutella* (1). The results showed 93.33% (14) of the fungal filtrates induced significant mortality, indicating their molluscicidal activity, with *Pochonia chlamydosporia* FCCUFG 100 and *Volutella aeria* FCCUFG 107 causing 100% mortality in all dilutions. These results reveal the potential of *Hypocreales* fungi from a Brazilian Cerrado cave as a promising approach for snail control.

## 1. Introduction

Fungi exist in many places worldwide and play important ecological roles in different biomes/ecosystems [1,2]. The Brazilian savanna (Cerrado) is the second largest biome in Brazil, covering more than 20% of the national territory with a rich speleological heritage (with around 11,000 caves) [3]. Studies have demonstrated the impact of fungi on various aspects of this biome, whether in underground environments [4,5] or associated with plants [6]. However, the gradual loss of territory has concerned researchers [7] and motivated studies on the fungi of this global biodiversity hotspot and their possible applications in society [8].

The complexity of caves has been observed to have a particularly close relationship between geodiversity and biodiversity [9]. This results in the formation of an ecosystem where the distinct geological characteristics of caves, such as rock formations, temperature, and humidity (abiotic factors), allow for a vast diversity of species and interactions between them (biotic factors). The current worldwide speleomycological estimate is that there are around 2000 fungal species that have already been reported in this ecosystem [4,10,11,12] and are transported from the external environment to the interior of the caves by wind, animals, among other factors [11,13].

The adaptive capacity of cave fungi is advantageous for developing bioactive compounds and their biotechnological applications [14]. Therefore, understanding the mechanisms by which fungi adapt to underground conditions can provide valuable information for biotechnological innovations, given the increasing number of fungi found in Brazil [15]. Fungi of the order *Hypocreales* also stand out for their remarkable morphological diversity and ability to establish diverse environmental interactions [16]. These interactions range from symbiosis with other organisms to pathogenic relationships and biological control [1,8,17]. In addition, they have significant biotechnological potential, encompassing a variety of bioactive compounds, including peptides with antimicrobial action [18,19,20,21], biopesticides [20,22,23,24,25,26], and medicinal properties [14].

Schistosomiasis is a neglected tropical disease caused by the *Schistosoma* parasite. The freshwater snails are the intermediate hosts that maintain the cycle. Among the freshwater snails naturally infected by *Schistosoma mansoni* in Brazil, *Biomphalaria glabrata* [27] stands out due to its wide geographical distribution, greater susceptibility to infection and effectiveness in transmitting the parasite [28,29]. The wide geographical distribution of gastropods is related to the high prevalence and incidence of the disease, reflecting its persistence in certain regions, and is related to sanitation infrastructure, water supply, sewage treatment, and solid waste management [29,30]. Thus, studies focused on intervention are important strategies against schistosomiasis [31]. In this regard, niclosamide has been the gold standard for combating schistosomiasis in endemic areas since the 1960s [32]. Nevertheless, it is expensive and can be toxic for non-target organisms [33]. The strategies established by resolution 71/313 of the United Nations reinforce implementing the 2030 Agenda for Sustainable Development [34,35]. Given the need to investigate new strategies for controlling *Schistosoma* intermediate hosts, it is crucial to seek solutions that offer greater affordability and specific control efficiency [36]. In this context, bioactive metabolites of fungal origin extracted from fungi show significant potential for environmentally sustainable and safe control compared to chemical alternatives. Using fungus-derived compounds is an advantageous and low-cost process that deserves further exploration [37]. These compounds also showed potential for the control of terrestrial [38,39] and aquatic [38,40,41] gastropods of medical or agricultural importance [42,43,44].

Fungal diversity in Cerrado caves has only recently begun to be studied [4,5], with this study being the first to investigate the molluscicidal activity of these cave fungi for the control of freshwater snails and considering this as another important topic for the One Health approach, the study of cave fungi and the investigation of their molluscicidal activity may provide new alternatives for the control of *Schistosoma* intermediate hosts, and for the discovery of new biomolecules. This study aimed to evaluate the molluscicidal potential of the filtered post-culture medium of *Hypocreales* fungi from a cave in the Brazilian Cerrado against the freshwater snails *B*. *glabrata*. The hypothesis that *Hypocreales* filtrates possess molluscicidal activity was tested, making it the first investigation into the molluscicidal properties of cave fungi. This study contributes to expanding our knowledge of the biotechnological potential of fungi and their application in snail control programmes.

## 2. Materials and Methods

### 2.1. Hypocreales Isolates

*Hypocreales* isolates were obtained from the working collection Coleção de Culturas de Fungos da Universidade Federal de Goiás (FCCUFG) housed at the Laboratório de Micologia (LabMicol) of the Instituto de Patologia Tropical e Saúde Pública (IPTSP), Universidade Federal de Goiás (UFG), Goiânia, Brazil. The *Hypocreales* fungi were isolated as airborne (n = 3) and from soil/sediment (n = 12) from the Lapa do Boqueirão cave (15°24′34″ S and 48°43′57″ W), located in the municipality of Vila Propício, Goiás state, an area of the Brazilian Cerrado in the central–west region of the country. The collection was conducted under the authorization of ICMBio/Cecav (SISBIO Number: 82254-1) [5]. This study was registered in the Sistema Nacional de Gestão do Patrimônio Genético (SisGen)/MMA/Conselho de Gestão do Patrimônio Genético (CGen) (registration number: ACD07BF). Fungal isolation was performed as described by Alves et al. [4] and Oliveira et al. [5].

### 2.2. Hypocreales Fungi Identification

The isolates (n = 15) were subcultured on potato dextrose agar (PDA) (Kasvi, Pinhais, PR, Brazil) and incubated at 26 °C for 7 days on a 12 h light and 12 h dark cycle to evaluate macroscopic (e.g., colour and texture) and micromorphological (e.g., conidiophores and conidia) characteristics based on the literature (e.g., [45]). For DNA extraction, we used 7-day-old cultures grown on a PDA using the Wizard^®^ Genomic DNA Purification Kit (Promega Corporation, Madison, WI, USA), following the manufacturer’s protocol. For DNA amplification, we used the primers ITS5 and ITS4 [46]. PCR, sequencing, sequence editing, and maximum likelihood (ML) phylogenetic analysis were carried out as described by Alves et al. [4]. DNA sequence alignment was constructed based on recent articles dealing with the *Hypocrales* genera included in our study (e.g., [47,48,49,50]). ML bootstrap support values equal to and greater than 70% were considered statistically significant and included near tree nodes. The sequences obtained in this study were deposited in GenBank (Table 1).

### 2.3. Fungal Filtrates

To obtain fungal filtrates from *Hypocreales* isolates, we followed the method by Siqueira et al. [51] with adaptations. Briefly, fungal isolates were grown on PDA at 26 °C for 7 days, and five discs (6 mm) of mycelium were inoculated into Erlenmeyer flasks containing 150 mL of potato–dextrose broth (PDB) (4% potato) with the pH adjusted to 7.2 ± 0.2. The flasks were incubated at 26 °C for 10 days in the dark, with continuous agitation at 120 rpm. After fermentation, the broth was vacuum filtered through a 0.22 µm pore polysulfone filter (Millipore^®^, Burlington, MA, USA). The resulting supernatants were centrifuged three times at 5 °C and 9000 rpm for 15 min using a refrigerated centrifuge. After this process, the fungal filtrates were stored at 5 °C and used within four days of filtration. They were diluted to 10% and 50% only on the day of the bioassay with the snails, using reconstituted water (294 mg/L CaCl_2_, 123.3 mg/L MgSO_4_, 63 mg/L NaHCO_3_, and 5.5 mg/L KCl) [52].

### 2.4. Snails

Adult *B. glabrata* were obtained and maintained at the Centro Multiusuário de Produção e Experimentação Animal (CMPEA—IPTSP—UFG), Goiânia, Brazil. They were kept under controlled conditions of temperature (26 ± 1 °C), water pH (7.0 ± 1), and photoperiod (12 h light/12 h dark), fed three times a week with fresh lettuce (*Lactuca sativa*), following OECD Guideline No. 243 [53]. Styrofoam plates were added to the aquariums to facilitate the collection of egg clutches. Nine days before the experiment, the egg clutches were scraped off the plates and kept in trays with dechlorinated water until hatching.

### 2.5. Acute Toxicity Tests with Newly Hatched Snails

The bioassay followed the methods described by Melo et al. [54] and Caixeta et al. [55] with adaptations. Newly hatched snails were distributed in 12-well microplates (Kasvi^®^). Each well contained 5 newly hatched snails with 3 mL of medium (n = 15 snails per replicate; n = 45 snails per experimental condition). The microplates were kept in a Biochemical Oxygen Demand (BOD) incubator (Solab Científica^®^, Piracicaba, SP, Brazil) under controlled conditions of temperature (26 ± 1 °C), humidity (70 ± 5%), and photoperiod (12 h light/12 h dark). Newly hatched snails were exposed to undiluted fungal filtrates (FFs) and two dilutions of the filtrates (FF10 = 10% and FF50 = 50%) for 96 h, under static conditions (i.e., without renewal of the medium). The negative control (NC) group was composed of reconstituted water [52], while the positive control was exposed to 0.05 mg/L niclosamide (Baylucide^®^, Bayer, Leverkusen, Nordrhein-Westfalen, Germany) [56]. In addition, to verify the potential effect of the culture medium, 3 additional replicates were exposed to PDB (4% potato, pH 7.2 ± 0.2). Mortality was assessed daily under a Zeiss Stemi 5080 (Oberkochen, Baden-Wurttemberg, Germany) stereomicroscope associated with a Zeiss Axiocam 150 colour camera. Snails with immobility, shell discolouration, visceral mass exposure, signs of necrosis, or the absence of a heartbeat were considered dead [54,55].

### 2.6. Statistical Analyses

The statistical analyses were performed in R software v. 3.5.2 [57], while graphs were performed in GraphPad Prism software v. 9.1.2 [58]. The survival analysis with the Log-Rank test a posteriori was performed to assess significant differences in the survival curve. Significant differences were considered at *p* < 0.05.

## 3. Results

### 3.1. Cave Hypocreales Fungi

**Figure 1:**
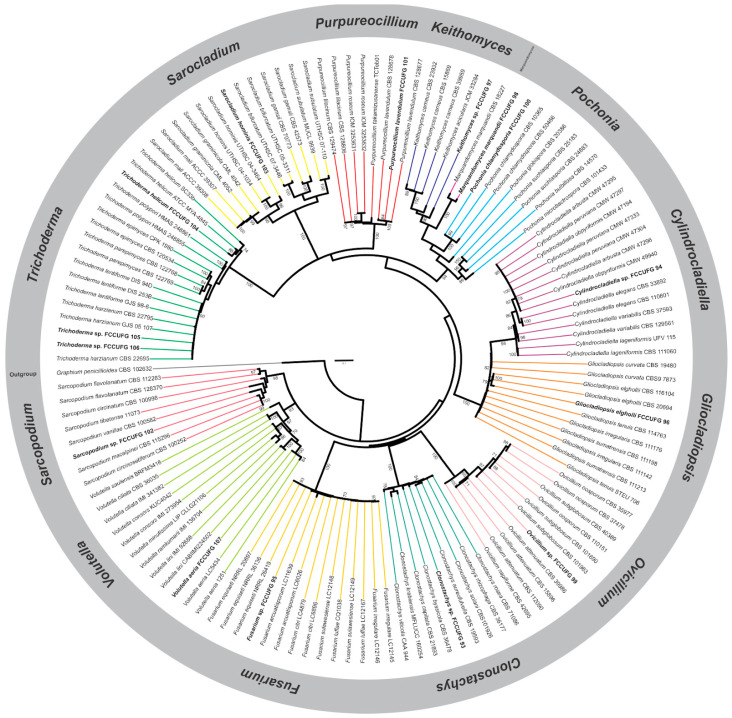
Maximum likelihood (ML) tree using ITS rDNA sequences representing 13 genera of *Hypocreales* fungi from a cave in the Brazilian Cerrado. Fungal isolates obtained in this study are in bold. Confidence values for ML-BS ≥ 70% are shown near the nodes, and the scale bar represents the expected number of changes per site. The tree was rooted to *Graphium penicillioides* CBS 102632 (*Microascales*).

Based on the macro- and micromorphological features and ML phylogenetic analysis of ITS rDNA sequences (Figure 1), the 15 isolates selected for this study were identified as belonging to 13 genera of the order *Hypocreales*: *Clonostachys* (1), *Cylindrocladiella* (1), *Fusarium* (1), *Gliocladiopsis* (1), *Keithomyces* (1), *Marquandomyces* (1), *Ovicillium* (1), *Pochonia* (1), *Purpureocillium* (1), *Sarcopodium* (1), *Sarocladium* (1), *Trichoderma* (3), and *Volutella* (1) (Table 1). Isolates of seven genera were identified at the species level (e.g., FCCUFG 104 = *T*. *helicum*, FCCUFG 103 = *S*. *hominis*, FCCUFG 101 = *P*. *lavendulum*, FCCUFG 98 = *M*. *marquandii*, FCCUFG 100 = *P*. *chlamydospora*, FCCUFG 96 = *G*. *elgholli*, and FCCUFG 107 = *V*. *aeria*) and other isolates were identified up to the genus level, which will be further investigated based on DNA sequences of different markers, as they were placed in a clade of highly related species/species complex or were considered putative new species.

### 3.2. Molluscicidal Activity

**Figure 2:**
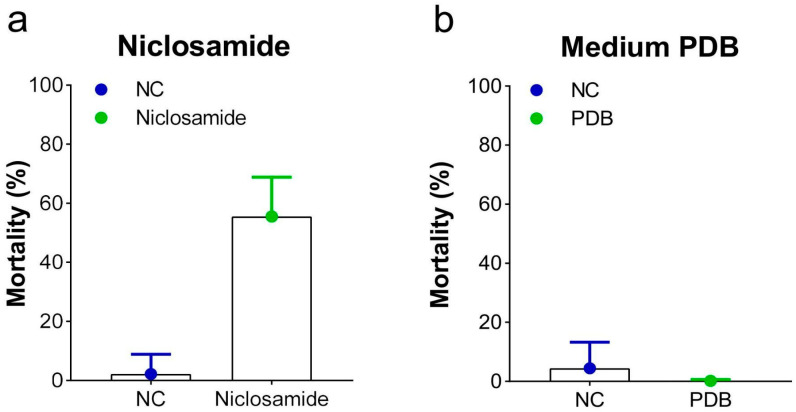
Mortality rates (means ± standard deviation) of *Biomphalaria glabrata* newly hatched snails exposed to (**a**) niclosamide at 50% lethal concentration (LC_50_) and (**b**) culture medium (PDB) after 96 h. NC = negative control group composed of reconstituted water.

The positive control (niclosamide) and the culture medium (PDB) groups validated the bioassay. The positive control resulted in a 50% mortality rate among the snails, while no significant difference in culture media compared to the negative control was observed (Figure 2).

**Figure 3:**
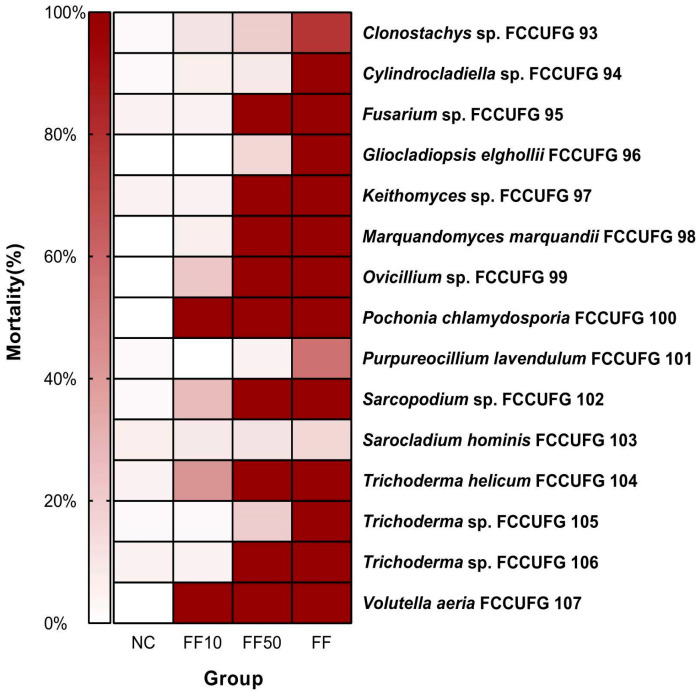
Cumulative mortality (%) of *Biomphalaria glabrata* newly hatched snails from the negative control (NC) and after exposure to filtrates diluted to 10% (FF10) and 50% (FF50) and undiluted fungal filtrates (FF) for 96 h. The experiment used fungal filtrates produced by isolates representing 13 genera of *Hypocreales* fungi from a cave in the Brazilian Cerrado.

**Figure 4:**
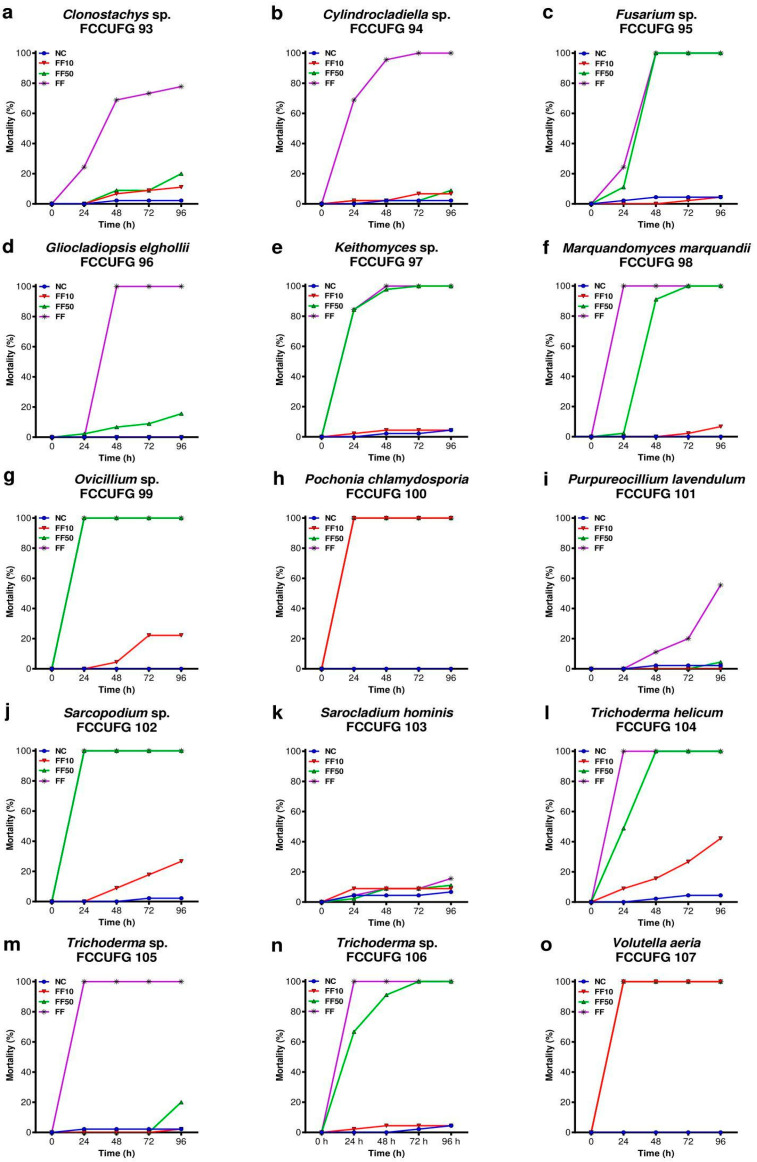
Cumulative mortality (%) of newly hatched *Biomphalaria glabrata* snails from the negative control group (NC) and exposure to filtrates diluted to 10% (FF10) and 50% (FF50) and undiluted fungal filtrates (FF) during 96 h. The experiment used fungal filtrates produced by isolates representing 13 genera of *Hypocreales* fungi from a cave in the Brazilian Cerrado. (**a**) *Clonostachys* sp. FCCUFG 93. (**b**) *Cylindrocladiella* sp. FCCUFG 94. (**c**) *Fusarium* sp. FCCUFG 95. (**d**) *Gliocladiopsis elghollii* FCCUFG 96. (**e**) *Keithomyces* sp. FCCUFG 97. (**f**) *Marquandomyces marquandii* FCCUFG 98. (**g**) *Ovicillium* sp. FCCUFG 99. (**h**) *Pochonia chlamydosporia* FCCUFG 100. (**i**) *Purpureocillium lavendulum* FCCUFG 101. (**j**) *Sarcopodium* sp. FCCUFG 102. (**k**) *Sarocladium hominis* FCCUFG 103. (**l**) *Trichoderma helicum* FCCUFG 104. (**m**) *Trichoderma* sp. FCCUFG 105. (**n**) *Trichoderma* sp. FCCUFG 106. (**o**) *Volutella aeria* FCCUFG 107.

**Figure 5:**
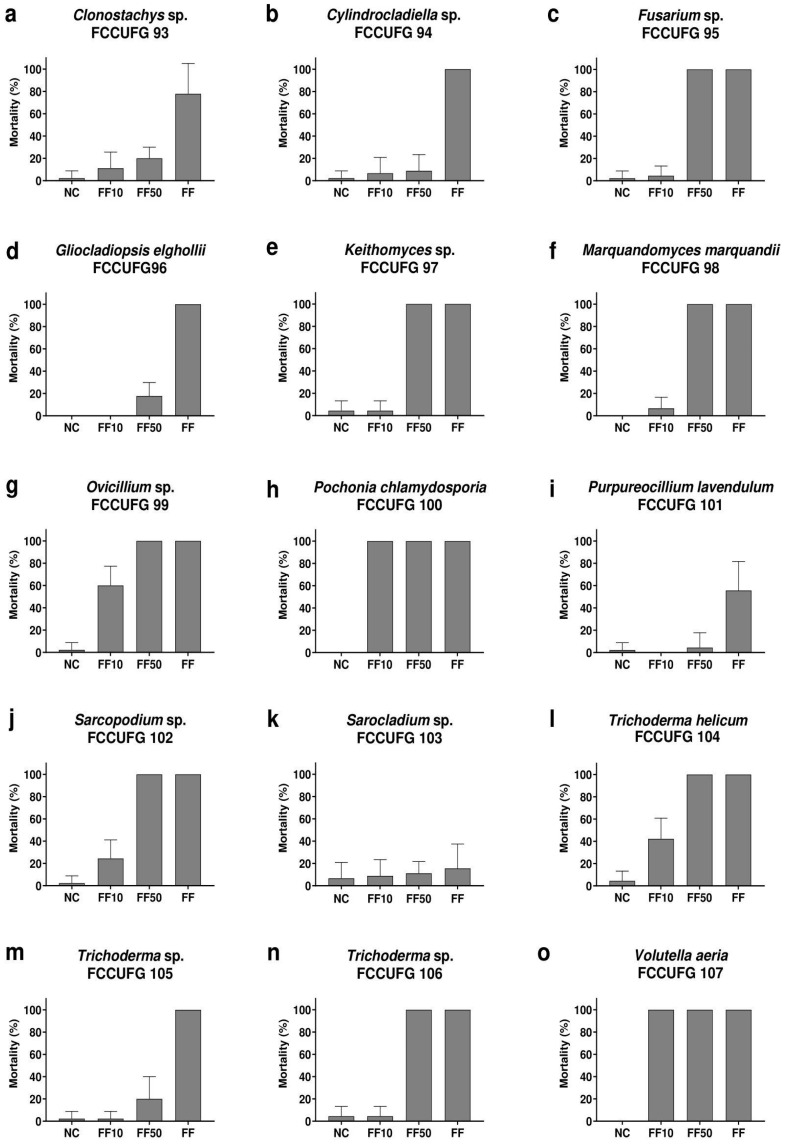
Mortality rate (means ± standard deviation) of newly hatched *Biomphalaria glabrata* snails. Negative control group (NC) and exposure to filtrates diluted to 10% (FF10) and 50% (FF50) and undiluted fungal filtrates (FF) during 96 h. The experiment used fungal filtrates produced by isolates representing 13 genera of *Hypocreales* fungi from a cave in the Brazilian Cerrado. (**a**) *Clonostachys* sp. FCCUFG 93. (**b**) *Cylindrocladiella* sp. FCCUFG 94. (**c**) *Fusarium* sp. FCCUFG 95. (**d**) *Gliocladiopsis elghollii* FCCUFG 96. (**e**) *Keithomyces* sp. FCCUFG 97. (**f**) *Marquandomyces marquandii* FCCUFG 98. (**g**) *Ovicillium* sp. FCCUFG 99. (**h**) *Pochonia chlamydosporia* FCCUFG 100. (**i**) *Purpureocillium lavendulum* FCCUFG 101. (**j**) *Sarcopodium* sp. FCCUFG 102. (**k**) *Sarocladium hominis* FCCUFG 103. (**l**) *Trichoderma helicum* FCCUFG 104. (**m**) *Trichoderma* sp. FCCUFG 105. (**n**) *Trichoderma* sp. FCCUFG 106. (**o**) *Volutella aeria* FCCUFG 107.

For the 15 fungal filtrates tested, 100% mortality was observed in 12 undiluted filtrates, 9 filtrates diluted to 50% (FF50), and 2 filtrates diluted to 10% (FF10) (Figure 3). For the lowest dilution (FF10), the filtrate from *P*. *chlamydosporia* FCCUFG 100 and *V*. *aeria* FCCUFG 107 were the ones that presented higher mortality rates, showing identical results, with 100% mortality in all dilutions tested after 24 h. The mortality curves for *B*. *glabrata* newly hatched snails over 96 h of exposure varied according to the exposure time and fungal filtrate tested (Figure 4). On the other hand, the mortality rate at the end of the experiment (Figure 5) showed the standard deviations for each treatment, highlighting the variability of the results. The filtrates of *P*. *chlamydosporia* FCCUFG 100 and *V*. *aeria* FCCUFG 107 showed equivalent results, with 100% mortality in all dilutions tested after 24 h (Figure 4o), making their molluscicidal action superior to all the post-culture fungal filtrates used. The third fungal filtrate with the most significant molluscicidal potential was *T*. *helicum* FCCUFG 104 (Figure 4l–n), which in FF50 caused the death of all the snails in 48 h, and 42.22% in FF10 in 96 h (Figure 4l). Among the other species, *Trichoderma* sp. FCCUFG 106 in FF50 caused 100% mortality in 72 h, and in FF10, mortality was similar to that of the negative control (Figure 4n), while *Trichoderma* sp. FCCUFG 105 in FF10 and FF50 was lethal for less than 20% of the newly hatched *B*. *glabrata* snails (Figure 4m), showing that it has the lowest molluscicidal potential among the genus.

## 4. Discussion

The search for alternative methods to reduce the number of schistosomiasis cases, a parasitic disease that affects millions of people in tropical and subtropical regions, primarily involves controlling the parasite’s intermediate host [36,59,60,61]. The use of alternative control methods is essential to help manage *B*. *glabrata* snails [36], and fungi and their metabolites could offer an important eco-friendly way to reduce the number of intermediate hosts of the aetiological agent of schistosomiasis [37,62,63]. Recently, it has been emphasized that fungi are important due to their high adaptability and biological and economic significance [64]. In this study, we analyzed the action of filtered post-culture medium from 15 *Hypocreales* fungi from a Cerrado cave in Brazil against *B*. *glabrata* newly hatched snails. The bioassay was validated by the outcomes observed in the positive control (niclosamide) and culture medium (PDB) groups. The 50% mortality observed in positive control was expected as the concentration tested corresponded to the lethal concentration 50 (LC50) for these newly hatched snails [56]. The exposure to the culture medium did not affect the mortality of *B*. *glabrata* newly hatched snails compared to the negative control group. Thus, the outcomes observed during the exposure to the different filtrates post-culture were related to the fungal metabolites and not the cultured medium. In addition, 14 fungal filtrates tested herein presented a high rate of molluscicidal activity. Among them, *V*. *aeria* FCCUFG 107 and *P*. *chlamydosporia* FCCUFG 100 stood out, delivering the best results, particularly at the lowest dilution (FF10) and within the shortest evaluation time (i.e., 24 h), consistently providing superior across all conditions analyzed.

In cave environments, fungi from the *Hypocreales* order, such as *Trichoderma*, *Fusarium*, and *Purpureocillium*, are among the most frequently cited [4,11,65] and play a fundamental ecological role in the decomposition of organic matter [66]. These fungi are economically and medically significant [67], although some of their species are reported to cause diseases in plants [68,69] and animals [70,71]. The secondary metabolism of *Hypocreales* fungi is vast, producing enzymes and other biomolecules (e.g., isocoumarins, sesquiterpenes, benzopyrans, and tetralones) [16,21,72,73,74] that are important in biocontrol [17,69]. However, their application as molluscicides remains underexplored, including investigating effective methods for controlling snails.

These biomolecules have great potential for application in medicine, especially in the pharmaceutical industry and biotechnological processes [14,75,76,77]. To the best of our knowledge, the action of secondary metabolites from cave fungi against snails has not yet been evaluated so far. Thus, this study is a pioneer in showing the potential molluscicidal application of these filtrates against newly hatched snails. The exploration of the biotechnological potential of cave fungi for controlling pathogens or their intermediate hosts could represent a valuable biological resource and deserve further investigation.

Based on the mortality rate observed in the lowest dilution tested (i.e., FF10) in the 15 *Hypocreales* fungi evaluated in the present study, *P*. *chlamydosporia* FCCUFG 100 and *V*. *aeria* FCCUFG 107 were those with the most significant molluscicidal potential, followed by *Trichoderma helicum* FCCUFG 104. Among the fungal filtrates analyzed, *P*. *chlamydosporia* stands out as the most studied fungus of the *Hypocreales* order, mainly regarding its nematophagous action [21,78,79]. In addition, it has been extensively researched for its nematicidal property against *Ancylostoma* sp. [78], *Meloidogyne* spp. [80], *Enterobius vermicularis* [81], and *Taenia saginata* [82], its insecticide property against *Spartocera dentiventris*, and its molluscicide property against the freshwater snails *Pseudosuccinea columella* [83] and *B*. *tenagophila* [84], indicating its potential use in controlling pests, vectors, and intermediate hosts. *Pochonia* and *Volutella* can produce a variety of metabolites with toxic activities, directly impacting the mortality of newly hatched snails. For example, some species of *Pochonia* are capable of releasing non-volatile mycotoxins (e.g., aurovertins) [85], volatile compounds [86], and enzymes (e.g., serine esterase) [87]. Some species of *Volutella* were able to produce terpenoids (e.g., voluhemines) [88,89], which inhibit sterol O-acyltransferase (SOAT) isoenzymes. As a consequence, it interferes with the formation of cholesterol esters and impairs lipid metabolism, which may contribute to cell failure in the snails.

Moreover, other fungi have also shown promising molluscicidal properties. In 2015, Duarte et al. [62] presented strategies for controlling *B*. *glabrata* egg clutches in two different devices, one with a water film in water agar medium and the other using a floating clay stone, using the fungi *Metarhizium anisopliae* and *Beauveria bassiana*, highlighting that the effectiveness of such control varies according to the fungal species, fungal propagules (with hyphae being more effective than conidia), and the environment in which they were applied. Other examples of the effectiveness of using fungi with molluscicidal action can be found in the work of Araújo et al. [90], who investigated the effect of usnic acid extracted from the lichen *Cladonia substellata* and proved the effectiveness of this substance in controlling *B*. *glabrata* egg masses and adults. In addition, another study by Altoé et al. [91] demonstrated the potential of silver nanoparticles obtained from the extract of the fungus *Monacrosporium thaumasium* to control *B*. *glabrata* egg masses, observing that these nanoparticles effectively inhibited the development of the embryos. This underscores the potential of several compounds originating from fungi in the effective management of different species of snails, elucidating the importance of identifying fungal substances capable of acting effectively in snail control.

Screening fungi found in caves to control gastropods represents a valuable biological resource, especially considering that most current research focuses on discovering molluscicides from plant extracts [92,93,94]. While the molluscicidal activity of plant extracts has been more extensively explored, fungi offer a promising alternative. Identifying bioactive compounds in fungal filtrates can reveal significant molluscicidal potential [37], particularly considering that their release of secondary metabolites may vary depending on cultivation conditions and fungal isolates (e.g., [95]). This potential is exemplified by the results obtained from the genus *Trichoderma* in the present study, which showed a significant variation in the mortality of *B*. *glabrata* snails exposed to fungal filtrates. While 100% mortality was observed in newly hatched snails exposed to the three undiluted *Trichoderma* filtrates within 24 h, the molluscicidal action varied between different strains. For instance, *T*. *helicum* FCCUFG 104 induced complete mortality in 48 h, while *Trichoderma* sp. FCCUFG 106 induced it in 72 h, and *Trichoderma* sp. FCCUFG 105 exhibited a much lower mortality rate at FF10 and FF50 dilutions.

As seen in our study, the variability in the snails’ responses to different dilutions of the fungal filtrate highlights the need for further research into its molluscicidal activity. This underscores the significance of screening fungal isolates within the same order, family, or genus [19,72], as observed in previous research [1,19,69]. This action encourages the study of mycodiversity in Brazilian Cerrado, emphasizing the necessity for more in-depth studies on the eco-friendly action of fungal compounds and their potential applications in molluscicide investigation.

In conclusion, the current study is a pioneer in analyzing the molluscicidal activity of the aqueous filtrate of *Hypocreales* fungi from a Brazilian savanna (Cerrado) cave against newly hatched *B*. *glabrata* snails. The initial hypothesis was accepted, demonstrating that *Hypocreales* filtered post-culture media containing fungal metabolites have molluscicidal activity against newly hatched *B*. *glabrata* snails. Among the fungal filtrates analyzed, *Pochonia chlamydosporia* FCCUFG 100 and *Volutella aeria* FCCUFG 107 stood out for causing 100% mortality of the newly hatched snails at the lowest dilution tested (FF10). Further studies should focus on *Pochonia chlamydosporia* FCCUFG 100 and *Volutella aeria* FCCUFG 107 filtrates, evaluating the yield of crude extract, the isolation and quantification of the compounds present in the filtrates, and testing other life stages of the snails. Overall, the current study can contribute to developing new fungi-based strategies to control snails of medical importance.

## Figures and Tables

**Table 1:** *Hypocreales* fungi from a Cerrado cave in Brazil with potential use against *Biomphalaria glabrata* newly hatched snails.

Taxa	Isolate	Substrate	GenBank Number (ITS)	BLAST Match (Taxon Name/ID%)
*Clonostachys* sp.	FCCUFG 93	Soil/sediment	PQ400040	*Clonostachys rosea* 100%
*Cylindrocladiella* sp.	FCCUFG 94	Soil/sediment	PQ400041	*Cylindrocladiella peruviana* 100%
*Fusarium* sp. (*F. incarnatum-equiseti* species complex)	FCCUFG 95	Air	PQ400042	*Fusarium* sp. 100%
*Gliocladiopsis elghollii*	FCCUFG 96	Soil/sediment	PQ400043	*Gliocladiopsis* sp. 100%
*Keithomyces* sp.	FCCUFG 97	Air	PQ400044	*Keithomyces* sp. 98.66%
*Marquandomyces marquandii*	FCCUFG 98	Soil/sediment	PQ400045	*Marquandomyces marquandii* 100%
*Ovicillium* sp.	FCCUFG 99	Soil/sediment	PQ400046	*Ovicillium* sp. 99.80%
*Pochonia chlamydosporia*	FCCUFG 100	Soil/sediment	PQ400047	*Pochonia chlamydosporia* 100%
*Purpureocillium lavendulum*	FCCUFG 101	Soil/sediment	PQ400048	*Purpureocillium lavendulum* 100%
*Sarcopodium* sp.	FCCUFG 102	Soil/sediment	PQ400049	*Sarcopodium macalpinei* 99.05%
*Sarocladium hominis*	FCCUFG 103	Air	PQ400050	*Sarocladium hominis* 97%
*Trichoderma helicum*	FCCUFG 104	Soil/sediment	PQ400051	*Trichoderma helicum* 99.60%
*Trichoderma* sp.	FCCUFG 105	Soil/sediment	PQ400052	*Trichoderma harzianum* 100%
*Trichoderma* sp.	FCCUFG 106	Soil/sediment	PQ400053	*Trichoderma harzianum* 100%
*Volutella aeria*	FCCUFG 107	Soil/sediment	PQ400054	*Volutella aeria* 100%

## Data Availability

The original contributions presented in this study are included in the article. Further inquiries can be directed to the corresponding author.
